# Recent Advances in Marine Algae Polysaccharides: Isolation, Structure, and Activities

**DOI:** 10.3390/md15120388

**Published:** 2017-12-13

**Authors:** Shu-Ying Xu, Xuesong Huang, Kit-Leong Cheong

**Affiliations:** 1 Guangdong Provincial Key Laboratory of Marine Biotechnology, STU-UNIVPM Joint Algal Research Center, Department of Biology, College of Science, Shantou University, Shantou 515063, China; l7syxu@stu.edu.cn; 2 Department of Food Science and Engineering, Jinan University, Guangzhou 510632, China; thxs@jnu.edu.cn

**Keywords:** marine algae, polysaccharide, extraction, characterization, structure-function relationship

## Abstract

Marine algae have attracted a great deal of interest as excellent sources of nutrients. Polysaccharides are the main components in marine algae, hence a great deal of attention has been directed at isolation and characterization of marine algae polysaccharides because of their numerous health benefits. In this review, extraction and purification approaches and chemico-physical properties of marine algae polysaccharides (MAPs) are summarized. The biological activities, which include immunomodulatory, antitumor, antiviral, antioxidant, and hypolipidemic, are also discussed. Additionally, structure-function relationships are analyzed and summarized. MAPs’ biological activities are closely correlated with their monosaccharide composition, molecular weights, linkage types, and chain conformation. In order to promote further exploitation and utilization of polysaccharides from marine algae for functional food and pharmaceutical areas, high efficiency, and low-cost polysaccharide extraction and purification methods, quality control, structure-function activity relationships, and specific mechanisms of MAPs activation need to be extensively investigated.

## 1. Introduction

Marine algae are the most abundant resources in the ocean. It is logical to consider that marine algae could be a key resource containing rich source of functional metabolites such as polysaccharides, proteins, peptides, lipids, amino acids, polyphenols, and mineral salts [1]. Marine algae or seaweeds can be classified into three major groups based on their pigmentation and chemical composition: (1) brown seaweed (Phaeophyceae); (2) red seaweed (Rhodophyceae), and (3) green seaweed (Chlorophyceae). There is a variety of seaweeds that have been used extensively in Asia as dishes or in foods such as soup, condiments, and salads [2]. In Asian countries, a diet rich in seaweed has consistently been linked to a lower incidence of chronic diseases such as cancer, cardiovascular, and heart diseases [1,3].

In recent years, numerous polysaccharides isolated from marine algae have attracted great interest in functional foods, pharmaceuticals, and cosmetic applications. Polysaccharides are a type of biomacromolecule that exist as cell wall structural components of marine algae. Polysaccharides from marine algae are often closely linked to pharmacological activities such as anticoagulant, antioxidant, antitumor, and immunomodulatory [4,5]. Usually, polysaccharides bioactivities are closely correlated with their chemical properties such as molecular sizes, types, and ratios of constituent monosaccharides, and features of glycosidic linkages [6]. A basic understanding of marine algae polysaccharides’ (MAPs) chemico-physical properties and biological activities are essential for successful polysaccharide application in functional foods and will help access to their multifunctional applications.

This review focuses on MAPs and presents an overview of their isolation, purification, and characterization in addition to biological activities with potential health benefits. The relationships between structure and activities are clearly analyzed.

## 2. Extraction of Polysaccharides from Marine Algae

MAPs exist as structural components of algal cell walls. Algae cell walls are structurally complex and heterogeneous [7], which comprise a fibrous skeleton and an amorphous embedding matrix [8]. The main structural elements of cell walls are polysaccharides. They are composed of neutral and/or acidic, linear, and mixtures of branched polysaccharides. These polysaccharides are generally extracted with hot water [9], which is a popular and convenient method; it can be used to easily extract polysaccharides from algae [10], but the drawbacks of this method are that it is time-consuming, has a requirement for high temperatures, and has a low extraction efficiency. In general, the extraction methods involve elimination of interfering substances (such as low molecular weight compounds, lipids and colored matter from algal sample) using a mixture of methanol/chloroform/water (4:2:1; *v*/*v*/*v*) [11].

In order to increase extraction efficiency, novel extraction techniques, including microwave-, ultrasonic-, and enzyme-assisted extractions, have recently been applied to MAP extraction. Among these techniques, microwave-assisted extraction is one of the most widely used methods for extracting polysaccharides from marine algae. Microwaves are coupled to both electrical and magnetic fields and thus represent electromagnetic energy over a spectral frequency ranging from 300 to 300,000 MHz. The microwave-assisted extraction parameters include microwave power, irradiation time, ratio of solid to liquid, and temperature which can be optimized using response surface methodology [12]. Microwave-assisted extraction has advantages of short extraction time, low energy, and low cost, but with higher extraction efficiency when compared with conventional method [13]. Yuan and Macquarrie demonstrated that extracting the fucoidan required a short extraction time (15 min) using the microwave-assisted extraction method, while the conventional extraction method took much longer (3 h) [14]. It was also expected, however, that by using microwave-assisted extraction technique, polysaccharides would undergo degradation. Tsubaki et al. reported that the molecular weights and viscosity of the extracted polysaccharides could vary according to the microwave processing temperatures [15]. It is assumed that the viscosity of the extracted polysaccharides decreased with increasing temperatures due to polysaccharide degradation. Therefore, the effects of the microwave parameters on the percentages of polysaccharide degradation, sulfate content, viscosity, monosaccharide composition, and molecular weights were also considered in microwave-assisted extraction [16].

Ultrasonic-assisted extraction is another advanced extraction method that has become increasingly popular for extraction of polysaccharides from marine algae materials [17]. The effect of ultrasonic-assisted extraction is attributed to the propagation of ultrasound waves, which can result in the cavitation phenomenon. The implosion of cavitation bubbles can lead to the diffusion through the cell walls. Water soluble polysaccharides will be released once the cell walls break down. Kadam et al. reported that laminarin from brown seaweeds were extracted by ultrasonic-assisted extraction. Ultrasound was found to give higher content of laminarin compared with conventional water extraction [18]. Rahimi et al. explored an ultrasonic-assisted extraction to obtain a maximum yield of 8.3% sulfated polysaccharides from green algae. The optimized parameters were an extraction temperature of 66 °C, extraction duration of 40 min and 50-fold water [19]. Ultrasound-assisted extraction allowed the reduction of extraction time and did not significantly affect the structure and molecular weight of alginates and carrageenans [20].

Enzyme-assisted extraction has aroused considerable interest because its hydrolytic action weakens or disrupts the cell wall structure and also breaks down complex interior storage compounds and, therefore, releases the intracellular polysaccharides [21,22]. The current enzyme-assisted extraction methods that are mainly applied use commercially available enzymes, such as Viscozyme, Cellucast, Termamyl, Ultraflo, carragenanase, agarase, amyloglucosidase, xylanase, Kojizyme, Protamex, Neutrase, Flavourzyme, and Alcalase [23,24]. Hardouin et al. demonstrated that the endo-protease treatments significantly increased the extraction yields of sulfated polysaccharides from algal samples [25]. Additionally, supercritical fluid extraction [26] and ionic liquids extraction [27] have also become an alternative, sustainable, green extraction techniques with high yields of polysaccharides from algal cell walls.

The ever growing demand for polysaccharides of marine algae will promote the continuous exploration of more convenient extraction methods. The novel extraction methods in recent years which include microwave-assisted extraction, ultrasound-assisted extraction, and enzyme-assisted extraction have the advantages regarding extraction time, low energy and less use of solvent, and therefore are considered as “green techniques”. Furthermore, the enzyme-assisted extraction is considered to be one of the most potential methods to extract the MAPs due to its high efficiency with mild reaction conditions.

## 3. Purification Procedure

**Figure 1:**
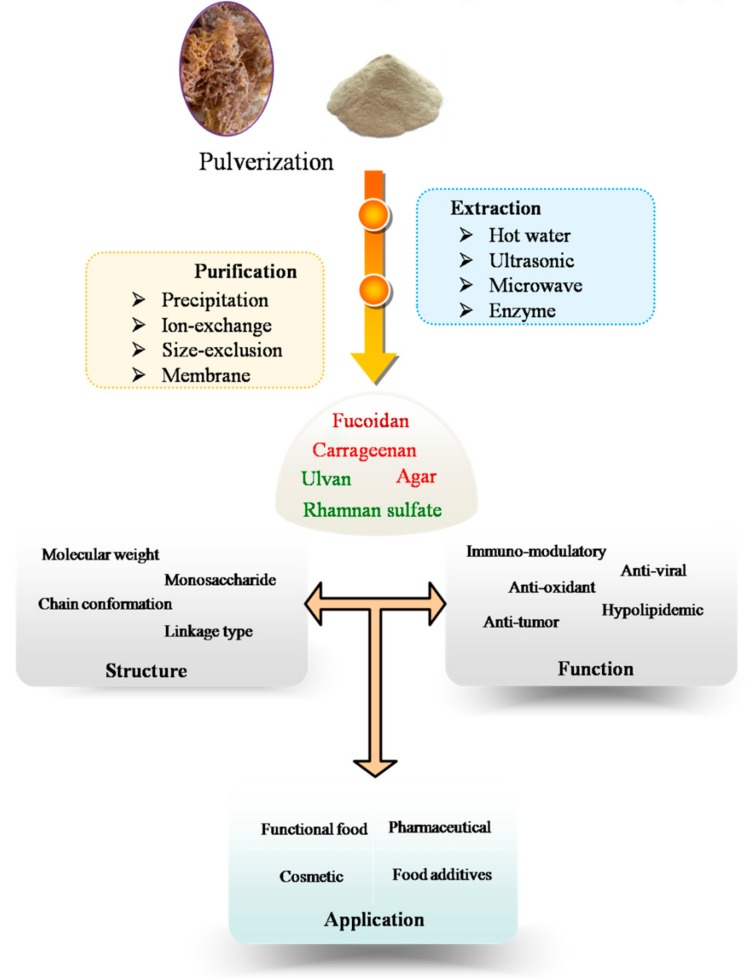
Schematic diagram of extraction, purification, characterization, biological activities, and applications of marine algae polysaccharides.

The procedure of MAPs extraction and purification include several steps that are summarized in Figure 1. Extracted polysaccharides are crude mixtures (with variable molecular weights, monosaccharides composition and sulfate content) when dissolved in water. Sometimes, they are together in the solution with proteins and low molecular weight compounds. These crude polysaccharide mixtures can be further purified using a variety of purification techniques, including ethanol precipitation, membrane separation, ion-exchange, size-exclusion, and affinity chromatographic methods. Ethanol precipitation is always used in the first steps of polysaccharides purification, which remove the low molecular weight impurities from the polysaccharides [28,29].

Membrane separation techniques which include diafiltration, ultrafiltration, reverse osmosis, and nanofiltration have attracted attention for the fractionation of polysaccharides from marine algae for their potential industrial uses [30]. Numerous types of membranes, with a series of molecular weight cut-off, are available on the market [31].

Polysaccharides were primarily purified using column chromatography, such as ion-exchange chromatography, size-exclusion chromatography and affinity chromatography. In general, ion-exchange chromatography is suitable for the separation of neutral/acidic polysaccharides from negatively charged polysaccharides via gradient salt elution or a change in pH [32,33,34]. Peng et al. fractionated the aqueous extracts of *Laminaria japonica* through a diethylaminoethyl (DEAE)-A25 anion-exchange column with gradient elution with 0.7–0.8 mol/L NaCl [35]. Size-exclusion chromatography is available for separation of polysaccharides from marine algae with different molecular weights or molecular sizes [36,37,38]. A sulfated polysaccharide from green algae was first purified through a Sephacryl S-300 HR size-exclusion column, and after enzymatic hydrolysis, three fractions of low molecular weight oligosaccharides were then collected from a Bio-gel P-4 size-exclusion column [39]. Recently, dye affinity chromatography is often used as powerful tool for MAPs purification. It exhibited a high ability to capture anionic polysaccharide selectively from crude extracts by immobilized thiazine dyes (e.g., toluidine blue and thionine acetate) [40]. Hahn et al. purified the fucoidan from brown algae by affinity chromatography on amino-derivatized Sepabeads [41].

The aqueous two-phase system is also available for the purification of polysaccharides. This liquid-liquid system typically uses polyethylene glycol and a saline solution to prepare the polysaccharides [42]. The aqueous two-phase system can be used together with a high-speed counter-current chromatography instrument [43].

A variety of purification approaches mentioned above could be chosen, however there are no standardized protocols for purification yet. The purification methodologies of MAPs used in different areas depend on the requirement of their purity and function. For functional food industry, the crude extracts of MAPs are mainly obtained from ethanol precipitation and scaled-up by membrane separation. The membrane separation is a favorable method used in food industry with advantages of being of low cost and without using reagents. Meanwhile, the application of size-exclusion chromatography and affinity chromatography are needed for pharmaceutical industry to obtain high purity and active fractions of MAPs.

## 4. Structural and Physical Properties of Marine Algae Polysaccharides

The monosaccharide composition, molecular weight, backbone, and structure-function relationship of polysaccharides from three main species of algae (red, brown and green algae) are summarized in Table 1.

Monosaccharide composition analysis remains one of the most important methods to profile composition of complex polysaccharides. A variety of chromatographic techniques have been used to separate and analyze monosaccharides [6]. Thin layer chromatography (TLC) has advantages in terms of simple sample preparation and a relatively low cost for detecting hydrolyzed monosaccharides from algae polysaccharides [44]. Foley et al. used the TLC method for preliminary investigation of monosaccharide composition of fucoidans [45]. High-performance liquid chromatography (HPLC) has been widely used to identify the constituent monosaccharides and molar ratios. Since monosaccharides have no ultraviolet (UV) absorbance, refractive index detectors (RID) [46] and evaporative laser scattering detectors [47] are usually used to detect monosaccharides composition of algae polysaccharide. The corona charged aerosol detector is a new type of detector that was developed for HPLC applications and has been used in recent years [48]. HPLC and capillary electrophoresis coupled with UV detectors are usually used to identify compounds with an absorption range of 190–380 nm. In this case, the reagents 1-phenyl-3-methyl-5-pyrazolone (PMP) and paminobenzoic ethyl ester are the most common derivatizing agents for monosaccharides under mild conditions. The monosaccharides obtained from hydrolysis of green algae polysaccharides were derivatized with PMP were found to compose of mannose (55.4 mol%), galactose (25.3 mol%), glucuronic acid (16.3 mol%), and arabinose (0.9 mol%) [49]. The high-performance anion-exchange chromatography (HPAEC), coupled with pulsed amperometric detection, was developed due to its high sensitivity and good resolution for detection of underivatized monosaccharides [50]. Products derived from a complete acid hydrolysis of alginate analyzed by the HPAEC technique showed that the composition of the two main uronic acids are glucuronic acid and mannuronic acid, with a ratio of 0.6 [51]. The molecular weight of polysaccharides are usually determined using size-exclusion chromatography, which is based on the molecular size as the basic principle [52]. High-performance size-exclusion chromatography (HPSEC) is always coupled with RID, which is a common and popular detector for the determination of polysaccharides. A series of dextran or pullulan standards with different molecular weights are used to calibrate the HPSEC system to determine the molecular weights of polysaccharide samples. Calibration curves using pullulan standards allowed to estimate the molecular weight of a sulfated polysaccharide purified from the green alga *Codium divaricatum* [53]. In recent years, the combination of HPSEC with a laser light scattering detector or a viscometer detector has been demonstrated to be a powerful and accurate method for determining the molecular weight, chain conformation and rheological properties of polysaccharides [54,55] without using a series of standards.

Mass spectrometry such as electrospray ionization mass spectrometry (ESI-MS) and matrix-assisted laser desorption ionization time-of-flight mass spectrometry (MALDI-TOF-MS) are also attractive alternative choices for obtaining molecular information about algal polysaccharides. A strong acid can almost completely degrade a polysaccharide into monosaccharides. However, low concentrations of acid (such as 0.05, 0.1 and 0.5 mol/L trifluoroacetic acid) can selectively hydrolyze or remove branched chains of one of the polysaccharides. Therefore, mild hydrolysis products could be directly analyzed by mass spectrometry even without purification. A MALDI-TOF MS/MS analysis of fucoidan fractions showed the presence of disaccharides α-l-Fuc*p*-2,4-di-OSO_3_^−^ as main component [56]. Using ESI-MS, Synytsya et al. characterized a mild hydrolysis fraction from a green algal sulfated polysaccharide. These fractions were identified as ulvanobiose (Xyl-RhaSO_3_Na) and disulfated disaccharide (GlcA-Rha) (SO_3_H)_2_ [49].

Nuclear magnetic resonance (NMR) spectroscopy is commonly used for polysaccharide structural analysis. NMR data (1D and 2D NMR) provide information about polysaccharide structure, including the monosaccharide composition, a presence of α- or β-type sugars, linkage features, and sequences of monosaccharide units [57]. A rhamnan-type polysaccharide, isolated from *Monostroma angicava*, was characterized by 1D and 2D NMR. This polysaccharide structure showed that two sulfate ester groups for every 10 →2)-α-*R*ha*p*-(1→ residues in the backbone [58]. Alginates from brown algae are linear polysaccharides with different ratios of mannuronic and glucuronic acids. This ratio varies from species to species of brown algae and could be determined by ^1^H NMR [51]. In order to investigate the effects of ultrasonic-assisted extraction on polysaccharide’s chemical structure, Youssouf et al. used ^1^H and ^13^C NMR to characterize polysaccharides extracted from *Sargassum binderi* and *Turbinaria ornata* and have found that ultrasound allowed for a reduction of extraction time without affecting the chemical structure of alginates and carrageenans [59].

## 5. Quality Control

**Figure 2:**
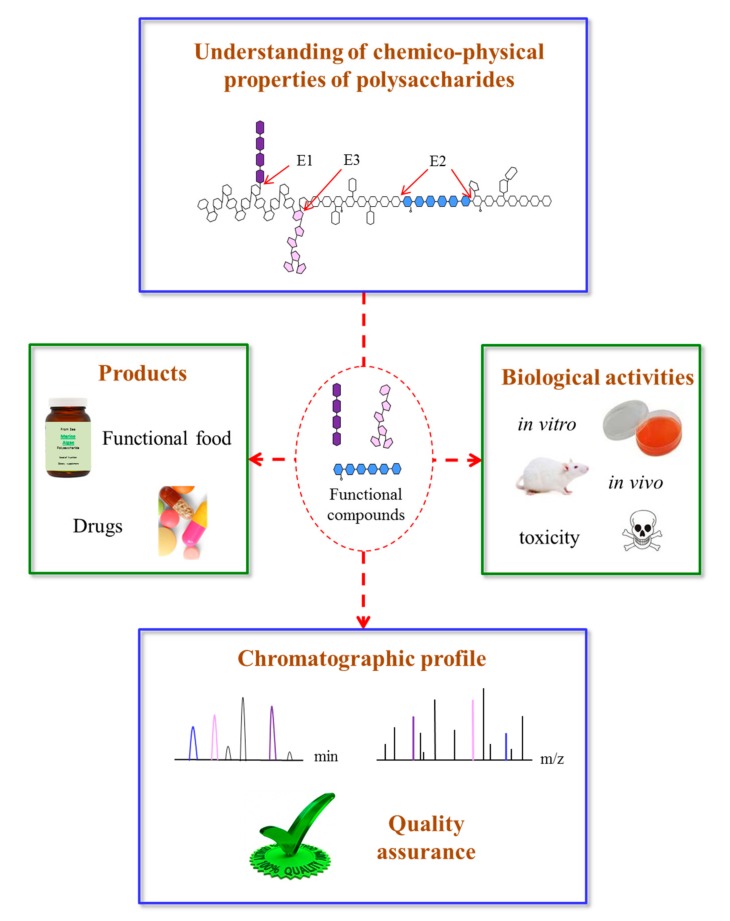
Schematic diagram of saccharide mapping (or enzymatic profile, E1–E3 are glycosidases) aiming to assess biological activities of marine algae polysaccharides. First, active compounds (color square) are digested by selected endo-glycosidases (E1–E3). Then biological activities are determined by in vitro and in vivo tests, as well as toxicity level. Finally, chromatographic profiles of active compounds are performed. This systematic approach could be used for quality assurance of developed products.

Recently, the quality control of polysaccharides has sparked the interest of many researchers, but research about this topic continues to be challenging because the structure of polysaccharides are complicated. In this sense, enzymatic polysaccharide profiles, or a method called saccharide mapping, show promising results in discriminating polysaccharides [6,78]. The schematic diagram of saccharide mapping, which plays a role in quality control of polysaccharides, is shown in Figure 2. First, a complex polysaccharide composed of different monosaccharides and may have a specific structure (color square) that is strongly related to their biological activity. This specific structure is then treated with a set of endo-glycosidases (E1–E3) to obtain enzymatic fragments. Their bioavailabilities are used for in vitro and in vivo tests as well as to determine their toxicity levels. Finally, chromatographic profiles are performed and can be used as markers. Using this strategy, quality control of these complex polysaccharides could be based on their functional groups. This could be helpful for controlling MAPs and related product quality.

## 6. Biological Activity of Polysaccharides from Marine Algae

In recent years, MAPs have been extensively studied due to the great interest in their biological activities, including antitumor, antiadhesive, antioxidant, antitoxin, immunomodulatory, anticoagulant, and anti-infection effects.

### 6.1. Immunomodulatory Activity

**Figure 3:**
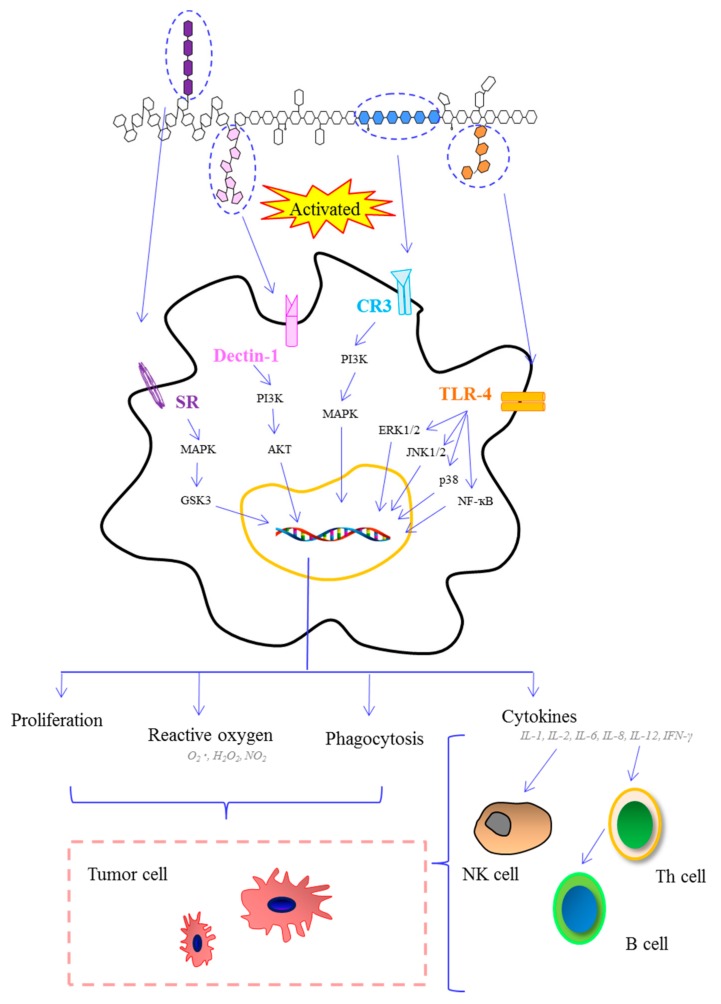
Schematic presentation of the immune system activated by marine algae polysaccharides after interaction of several molecular events. (Akt: protein kinase B; CR3: complement receptor 3; ERK1/2: extracellular signal regulated kinase 1/2; GSK3: glycogen synthase kinase 3-β; IFN: interferon; IL: interleukin; JNK1/2: c-Jun N-terminal kinase 1/2; MAPK: mitogen-activated protein kinase; PAK: p21-activated kinase; PI3K: phosphatidylinositol-3 kinase; SR: scavenger receptor; TLR-4: toll-like receptor 4; TNF-α: tumor necrosis factor-α).

The immune system consists of two types of immunity (innate and adaptive) and defends against foreign or potentially dangerous invaders [79]. MAPs can directly or indirectly interact with the immune system and trigger several signal pathways, which lead to immune system activation (Figure 3).

Macrophages are one of the most important types of cells of the innate immune system. Macrophages’ primary role includes phagocytic cell actions, antigen-presenting cell functions, and interaction with T lymphocytes in order to modulate the adaptive immune response. A polysaccharide from *Porphyra haitanensis* can increase phagocytosis in RAW264.7 macrophages. Meanwhile, this polysaccharide induced nitric oxide production through the Janus kinase (JAK2) and the Jun N-terminal kinase (JNK) signaling pathways in RAW264.7 macrophages [80]. A polysaccharide extracted from a brown alga *Hizikia fusiforme* showed the ability to increase nitric oxide (NO) production and inducible nitric oxide synthase (iNOS) expression in RAW264.7 macrophages [68]. A polysaccharide extracted from *Laminaria japonica* exhibited significant stimulation of macrophages and enhanced production of cytokines, such as tumor necrosis factor (TNF)-α, interleukins (IL)-1β, IL-6, and IL-10. This polysaccharide has positive effects on the phosphorylation of extracellular signal regulated kinase (ERK1/2), JNK1/2, and P38 [81].

Dendritic cells maturation play an important role in the function of innate and adaptive immune systems. Recent studies have shown that the fucoidan from *Fucus evanescens* induced stimulation and maturation of dendritic cells [82]. This study suggested that fucoidan-induced maturation of dendritic cells was mediated by TNF-α production and involved signal transduction through mitogen-activated protein kinase p38, phosphoinositide-3 kinase, and glycogen synthase kinase 3. Jeong et al. reported that fucoidan from marine algae has cytoprotective effects against 5-fluorouracil on dendritic cells in viability and cell size. This research suggested that fucoidan could maintain cancer patients’ immunity [83]. Natural killer (NK) cells also plays an important role in immuno-modulatory activity based on their ability to secrete cytokines, and home to lymph nodes and tissues, and their expansion in humans during increased immune tolerance [84]. Fucoidan obtained from *Ascophyllum nodusum*, *Macrocystis pyrifera*, *Undaria pinnatifida*, and *Fucus vesuculosus* promoted mouse NK cells activation. Among them, the fucoidan from *Undaria pinnatifida* showed the strongest effects as a result of NK cells expansion [85]. Sulfated polysaccharides from marine materials can prevent the adhesion of *Helicobacter pylori* and reduce biofilm formation. Besednova et al. speculated that the effect of algal polysaccharides on the infectious process caused by *Helicobacter pylori* was related to their actions on innate and adaptive immunity cells [86]. There are no reports about the toxicity of MAPs, and any undesirable effects of overdose and sensitivity to polysaccharides are still unknown. Toxicological tests are needed to examine any potentially harmful effects of MAPs on the human cells.

### 6.2. Antitumor Activity

Recent studies have demonstrated that MAPs possess significant antitumor activity both in vitro and in vivo. The antitumor mechanism of MAPs is believed to be due to stimulation of cell-mediated immune responses [87]. For example, polysaccharides from *Sargassum fusiforme* significantly inhibited the growth of human HepG2 cell-transplanted tumor in nude mice. It showed effective antitumor activity as a result of either directly attacking the cancer cells or enhancing the host’s immune function [88]. An antitumor polysaccharide purified from brown algae had a chemical structure that was identified as α-1,3-Fuc backbone. These fucoidans showed selective antitumor activity against different types of cancer cells, which were dependent on their unique structures [89]. Antitumor activity of fucoidans has been reported to be closely related to their sulfate content and molecular weights. The fucoidan isolated from *Eisenia bicyclis* demonstrated significant antitumor activity against the colon cancer DLD-1 and melanoma SK-MEL-28 cell lines. This purified fucoidan contained only fucose and had a high sulfate content (32.3%) [33]. Daily consumption of marine algae rich in fucoidans has been suggested as a reason for the reduction in postmenopausal breast cancer [90]. Therefore, marine algae polysaccharides can be used as functional ingredients in functional foods in order to potentially reduce tumor formation in the human body.

### 6.3. Antiviral Activity

In recent years, the constant outbreak of emerging or reemerging viral diseases has been detrimental to human health. Despite comprehensive studies for suitable vaccines and treatments against viral infections over the past half century, several infections such as human immunodeficiency, herpes simplex, hepatitis C, respiratory syncytial, and dengue viruses have afflicted a substantial proportion of the world’s population. These viruses attach to cells by an interaction between the envelope glycoprotein C and the cell surface heparin sulfate [91]. However, MAPs may block viral interactions with the cells based on formation of similar complexes. This raises the possibility of the application of MAPs in antiviral therapy.

Dinesh et al. reported that fucoidan fractions isolated from *Sargassum swartzii* are proficient in reducing HIV-1 p24 antigen levels in peripheral blood mononuclear cells (PBMC) and reversing transcriptase inhibition activity [92]. It was also reported that galactan sulfate isolated from *Agardhiella tenera*, displayed effective control against HIV-1 and -2. The galactan sulfate can block viral adhesion to cells [93]. Bandyopadhyay et al. reported that xylogalacto fucan and sulfated polysaccharides extracted from *Sphacelaria indica* had high antiviral activity against herpes simplex viruses. The study suggested that antiviral activity was dependent on the polysaccharide sulfate content [94].

### 6.4. Antioxidant Activity

Reactive oxygen species (ROS) such as hydrogen peroxide, superoxide anion, and hydroxyl radical are derived from the metabolism of molecular oxygen and can cause extensive damage to cell structures and tissues that eventually lead to various disease conditions [95], especially aging, cancer, heart, and neurodegenerative diseases. MAPs exhibit obvious antioxidant activity, and can be used to reduce oxidative damage to human body. Sousa et al. demonstrated that a polysaccharide fraction from marine alga *Solieriafili formis* had definite antioxidant activity. It displayed a dose-dependent 2,2-diphenyl-1-picrylhydrazyl (DPPH) radical scavenging effect of 1.77 mg/mL [96]. Some reports have demonstrated that the antioxidant activity of MAPs can be enhanced by decreasing their molecular weights. For example, degraded low molecular weight polysaccharide fragments from marine algae showed higher antioxidant capacity. They exhibited strong DPPH and •OH radical scavenging activity in addition to lipid peroxidation inhibition [97]. Jiménez-Escrig et al. isolated a polysaccharide from *Saccharina latissima*, which showed scavenging activity toward the radical 2,2′-azinobis-(3-ethylbenzothiazolin-6-sulphonate) [98]. This study suggested that acid-soluble fractions containing fucans showed higher antioxidant activity than those containing alginate and laminaran in *Turbinaria conoides* seaweed. A polysaccharide from *Sargassum fusiforme* showed protective effects on cyclophosphamide-induced immune suppressed mice. This polysaccharide caused significant improvements in superoxide dismutase and glutathione activities [99]. Polysaccharide fractions with a molecular weight of 2–4 kDa, isolated from *Hizikiafusi formis,* were shown to significantly improve the state of the liver in mice exposed to CCl_4_ [100]. Chitin is the most abundant amino polysaccharide, and is easily obtained from cell walls of Coralline algae [101]. Chitin showed significant antioxidant activity [102], and could be used as a thickener in cosmetics for skin protection.

### 6.5. Hypolipidemic Activity

A seaweed-based diet could prevent hyperlipidemic atherosclerosis by altering cholesterol absorption and metabolism, thereby decreasing plasma triglycerides (TG), overall cholesterol, and low density lipoprotein (LDL) cholesterol levels in hyperlipidemic mammals [3].

Sulfated polysaccharides from the green algae can be used as potent antihyperlipidemic agents due to their capacity to reduce apolipoprotein B100 secretion and lipid synthesis in HepG2 cells [103]. Zha et al. reported that crude polysaccharides from *Laminaria japonica* at a dose of 400 mg/kg/day caused a reduction in total serum cholesterol, TG, high density lipoprotein (HDL)-cholesterol, and LDL in serum [104]. In vivo administration of the sulfated polysaccharides from *Cystoseira crinite* to high-fat-diet rats led to a notable decrease in blood LDL and TG levels, but an increase in HDL levels [105]. Cao et al. reported that porphyran from *Pyropia yezoensis* at a dose of 200 mg/kg/day can decrease the percentage of body weight gain and serum lipid profiles of mice, similar to the effect of a hypolipidemic drug [106]. This study also suggested that dietary porphyran can alleviate liver damage induced by a high-fat diet. The acetylation of ulvans from *U. pertusa* by acetic anhydride showed higher antihyperlipidemic activity than natural ulvans, especially with regard to causing a decrease in TG and LDL-cholesterol levels [39].

## 7. Future Perspective

Marine algae have the advantages of strong adaptability and high yield, which are also considered as the most important source of many biologically active compounds, therefore it is of great significance to make better use of marine algae resources. Polysaccharides are one of the main components of marine algae and show significant biological activities. MAPs show promise for use in a wide range of functional foods and pharmaceutical products. However, there are still some weaknesses in the current study of MAPs. Although novel extraction techniques such as microwave-, ultrasonic-, and enzyme-assisted extraction methods show relatively high extraction efficiencies, they also have the advantages of simple operation, low cost and being environmentally friendly, these extraction techniques are limited to the research in the lab for MAPs extraction, there is still no research on an industrial scale. Furthermore, there are still no reports about MAPs quality control or chromatographic profiles. It is necessary to develop a simple and reliable method for MAPs quality control, which would be significant for improving the quality and performance of functional foods and pharmaceutical products. In addition, structurally well-defined polysaccharides from marine algae are of great importance for revealing their structure-bioactivity relationships. The chemico-physical properties, biological activities, and molecular mechanisms for MAPs also need to be extensively investigated.

## Figures and Tables

**Table 1:** The monosaccharide composition, molecular weight, backbone, polysaccharide type, and structure-function relationship of polysaccharide derived from three main species of algae (red, brown and green algae).

Species	Polysaccharide Type	Molecular Weight (Da)	Monosaccharide	Backbone	Biological Activities	Reference
**Red algae**	-	-	-	-	-	-
*Mastocarpus stellatus*	Carrageenan	1248 k	Gal:Glc:Xyl:Man = 87.8:5.4:4.4:2.4	β-1,3-Gal and α-1,4-Gal	Anticoagulant	[59,60]
*Chondrus armatus*	Carrageenan	88 k	Gal	β-1,3-Gal and α-1,4-Gal	Antiviral	[61]
*Nemalion helminthoides*	Sulphated mannan	43.8 k	Man:Xyl:Sulphate = 1:0.01:0.64	α-1,3-Man	Immunomodulatory	[62]
*Ahnfeltiopsis flabelliformis*	Sulphated galactan	-	Gal:3,6-AnGal:Glc:Xyl:SO_3_Na = 34.9:15.0:2.0:2.1:18.7	β-1,3-Gal and α-1,4-Gal	Anticoagulant	[63]
*Porphyra haitanensis*	porphyran	277 k	Gal	β-1,3-Gal	Antitumor	[64]
*Gracilaria fisheri*	Sulphated galactan	-	Gal	β-1,3-Gal and α-1,4-Gal	Antioxidant	[65]
*Cryptonemia seminervis*	Sulphated galactan	51.6 k	Gal, trace in Glc, Ara	β-1,3-Gal and α-1,4-Gal	Anti-metapneumovirus	[66]
*Gelidium crinale*	Sulphated galactan	300–600 k	Gal	α-1,3-Gal and α-1,4-Gal	Antiinflammatory	[67]
**Brown algae**	-	-	-	-	-	-
*Alaria marginata*	Galactofucan	-	Fuc:Gal:Xyl = 47.5:47.3:5.2	→3)-α-l-Fuc-(2,4-SO_3_^−^)-(1→	Anticancer	[32]
*Hizikia fusiforme*	-	-	Fuc:Gal:Xyl:Glc = 1.00:0.50:0.24:0.21	-	Immunomodulatory	[68]
*Cystoseira sedoides*	Fucoidan	642 k	Fuc and Uronic acid	α-1,3 or α-1,4-Fuc	Antiinflammatory	[69]
*Coccophora langsdorfii*	Fucoidan	-	Fuc	α-1,3 and α-1,4-Fuc	Anticancer	[70]
*Eisenia bicyclis*	Laminaran	19–27 k	Glc	β-1,3 and β-1,6-Glc	Anticancer	[71]
*Scytothamnus australis*	Sulphated fucan	-	Fuc:Xyl:Glc = 40.8:1.5:1	α-1,3-Fuc	Anti-HSV1	[72]
*Sargassum fusiforme*	Laminaran	27.6 k	Glc:Gal = 1.13:0.38	β-1,3-Glc, β-1,6-Glc	-	[73]
*Laminaria japonica*	Laminaran	-	Man:Ara:Glc:Gal:Fuc = 3.27:8.61:4.23:12.12:46.93	-	Antioxidant	[74]
**Green algae**	-	-	-	-	-	-
*Enteromorpha linza*	Rhamnan sulphate	108.4 k	Rha:Xyl:Man:Glc:Gal = 3.6:1.0:0.31:0.28:0.19	1,4-Rha	Antioxidant	[75]
*Codium divaricatum*	Sulphated galactan	37.9 k	Gal:Glc = 97.8:2.16	1,3- β-Gal	Anti-coagulant	[53]
*Capsosiphon fulvescens*	Ulvan	-	Rha:Xyl:Man = 45.0:44.1:10.2	4)-β-Xyl-(1→4)-α-Rha-(1→	Anticoagulant	[49]
*Ulva armoricana*	Ulvan	140–500 k	Rha:Gal:Glc:Xyl = 40.0:6.7:26.2:4.4	-	Antiviral	[25]
*Ulva pertusa*	Ulvan	28.2 k	-	-	Antiradiation	[76]
*Monostroma angicava*	Rhamnan sulphate	88.1 k	Rha	α-1,2-Rha, α-1,3-Rha	Anticoagulant	[58]
*Gayralia oxysperma*	Rhamnan sulphate	109 k	Rha:Xyl:Glc = 76.0:17.3:4.4	α-1,3-Rha	Antitumor	[77]

Gal, galactose; Glc, glucose; Xyl, xylose; Man, mannose; 3,6-AnGal, 3,6-anhydro-d-galactose; Ara, arabinose; Fuc, fucose; Rha, rhamnose.
